# Evidence-based review of oral traditional Chinese medicine compound recipe administration for treating weight drop-induced experimental traumatic brain injury

**DOI:** 10.1186/s12906-016-1076-2

**Published:** 2016-03-09

**Authors:** Bo Yang, Zhe Wang, Chenxia Sheng, Yang Wang, Jing Zhou, Xin-gui Xiong, Weijun Peng

**Affiliations:** Institute of Integrated Medicine, Xiangya Hospital, Central South University, Changsha, 410008 China; Department of Integrated Chinese and Western Medicine, The Second Xiangya Hospital, Central South University, No.139 Middle Renmin Road, Changsha, Hunan 410011 P.R. China

**Keywords:** Meta-analysis, Systematic review, Traumatic brain injury, Traditional Chinese medicine compound recipe

## Abstract

**Background:**

Recently, a number of studies conducted and published in China have suggested that traditional Chinese medicine compound recipe (TCMCR) may be beneficial in the treatment of experimental traumatic brain injury (TBI). In this study, we conducted a systematic review and meta-analysis of the efficacy of TCMCR in TBI model with weight drop method to provide robust evidence on the effects of TCMCR and to determine whether TCMCR can be recommended for routine treatment or considered as a standard treatment for TBI.

**Methods:**

We identified eligible studies by searching five electronic databases on April 1, 2014, and pooled the data using the random-effects model. Results were reported in terms of standardized mean difference (SMD). We also calculated statistical heterogeneity, evaluated the studies’ methodological quality and investigated the presence of publication bias.

**Results:**

Totally, 187 relevant publications were searched from databases, 25 of which met our inclusion criteria. The overall methodological quality of the most studies was poor, and there was evidence of statistical heterogeneity among studies along with small-study effects. Meta-analysis showed statistically significant effects indicating that TCMCR has a beneficial effect on TBI.

**Conclusions:**

Despite the limitations, we concluded that TCMCR may reduce brain water content, improve BBB permeability, and decrease TNF-α/NO expression after experimental TBI in terms of overall efficacy. However, our review also indicates that more well-designed and well-reported animal studies are needed.

## Background

Traumatic brain injury (TBI) remains the leading cause of long-term disability in individuals under 35 years worldwide [1], severely affects the quality of life of surviving patients and brings a significant social and economic burden. However, there is currently no effective pharmacological interventions options for TBI [2]. Because research aimed at therapy development has focused almost exclusively on single therapies, all of which have failed in multicenter clinical trials [3]. Fortunately, the focus of research has recently shifted to modify multiple targets, either through combination therapies or through the use of single agents that modulate multiple key secondary events following TBI [4, 5].

For thousands of years, traditional Chinese medicine (TCM) has been widely practice in China, and holds a key role in maintaining the health of the Chinese population, merits far greater attention from researchers because of its diverse pharmacological functions and targets, which can provide improved treatment of complex diseases by its ability to aim at several targets simultaneously [5]. The traditional Chinese medicine compound recipe (TCMCR), the main form of TCM drug treatment, may represent an ideal source for developing safe and effective agents for TBI treatment because it contains ≥2 Chinese herbs, and therefore more closely conforms to TCM theories and more accurately reflects the characteristics of TCM than does the administration of a single herb [6]. Recently, a number of studies conducted and published in China have suggested that TCMCR may be beneficial in TBI treatment and rehabilitation [7–11]. However, it is uncertain whether robust evidence exists on the effects of TCMCR or whether TCMCR can be recommended either for routine treatment or considered as a standard treatment for TBI. Moreover, a systematic review and meta-analysis of the efficacy of TCMCR in treating TBI has not yet been investigated in experimental animal studies.

Therefore, the primary aim of the present study was to conduct a systematic review and meta-analysis to investigate in an unbias manner whether the evidence from experimental studies indicated a beneficial effect of TCMCR in the treatment of TBI in animal models.

## Methods

### Literature search

All studies reporting the efficacy of TCMCR in animal TBI models prior to April 1, 2014 were included. Articles were searched in the following databases: PubMed, ScienceDirect, CNKI, Wan-Fang Data, and Vip. The key search terms are summarized in Table 1 and were kept broad to capture all potentially relevant articles. In addition, the reference lists of all relevant articles were searched for further relevant publications.

**Table 1:** Key search terms used in database searches

Traumatic brain injury	traditional Chinese medicine compound recipe
Traumatic brain injury	traditional Chinese medicine compound recipe
Traumatic brain injuries	traditional Chinese medicine recipe
Head injury	traditional Chinese herb medicine
Head injuries	traditional Chinese medicine
Brain injury	Chinese herb medicine
Brain injuries	Chinese medicine
Injury brain	herb medicine
Injuries brain	TCMCR
Head trauma	TCM
TBI	

### Study selection

Three investigators assessed the records to assess for eligibility based on title, abstracts. The copies of all relevant articles were obtained and further assessed whether each met the prespecified inclusion criteria, the details of which are presented in Table 2. Disagreements among investigators were resolved by consensus after discussion.

**Table 2:** Criteria for the inclusion/exclusion of studies

Inclusion criteria	Exclusion criteria
1. Published in peer-viewed journal	1. Non-published studies and dissertations
2. Was published in English or Chinese	2. No control group
3. TCMCR was administered orally	3. TCMCR administered in other methods (e.g. Intraperitoneally, subcutaneously, etc.)
4. Experimental TBI was induced in rodents	5. Examined other types of animals (e.g. sheep, cat, dog etc.)
5. Had a TBI treatment group that was treated with TCMCR and TBI control group that was administered a placebo following injury	6. Administration of traditional Chinese injection or a single Chinese herb in the treatment group
6. Investigators employed weight-drop methods to induce brain trauma	7. Involved non-impact (e.g. cortical ablation) or penetrating TBI. (e.g. missile-induced TBI)
	8. Duplicate publications

### Data extraction

Two investigators independently extracted details of each included studies including the animal species used, type of TBI model, treatment groups, time/dose of drug administration, anesthetic used, and the main outcomes. Information on sample sizes and substances used as experimental and control treatments was also extracted. Disagreements were resolved through consultation with a third party author.

When sufficient data were not available, authors were contacted and requested to provide missing data. The digital ruler software was used to estimate numerical values from the graphs, if no reply was received. And the study was excluded from the meta-analysis, when the required data were not obtainable.

### Study quality

The methodological quality of each included studies was assessed based on a 10-point quality checklist modified from the CAMARADES study as previously described, with minor modifications [12, 13], comprising (1) publication in a peer-reviewed journal; (2) random group allocation; (3) blinded induction of brain injury; (4) blinded assessment of outcome; (5) monitoring of physiological parameters including temperature; (6) sample size calculation; (7) compliance with animal welfare regulations; (8) avoidance of anesthetics with marked intrinsic neuroprotective properties (ketamine); (9) statement of potential conflicts of interest; (10) use of accurate/suitable/adequate animal models.

One point was given for written evidence of the quality criteria.

### Statistical analysis

Data were processed as described previously [14]. Briefly, for the meta-analysis, results were calculated as standardized mean difference (SMD), and 95 % confidence intervals (CI) with random-effects model to avoid heterogeneity were used to assay differences of the global estimate effect [13]. The Cochran’s *Q*-statistic was used to assess within- and between-study variation or heterogeneity [15, 16]. Heterogeneity was quantified with the *I*^2^ metric, with higher values denoting a greater degree of heterogeneity. *I*^2^ values ≤ 50 % indicate acceptable heterogeneity among studies [17]. For studies comparing different doses and/or timing of drug administration with a single control group, we pooled data from all experimental groups for comparison with the control group. The possible publication bias was assessed using funnel plots and Egger’s tests. [18]. All statistical analyses were performed using Stata software (version 12.0).

## Results

### Study selection

**Fig. 1:**
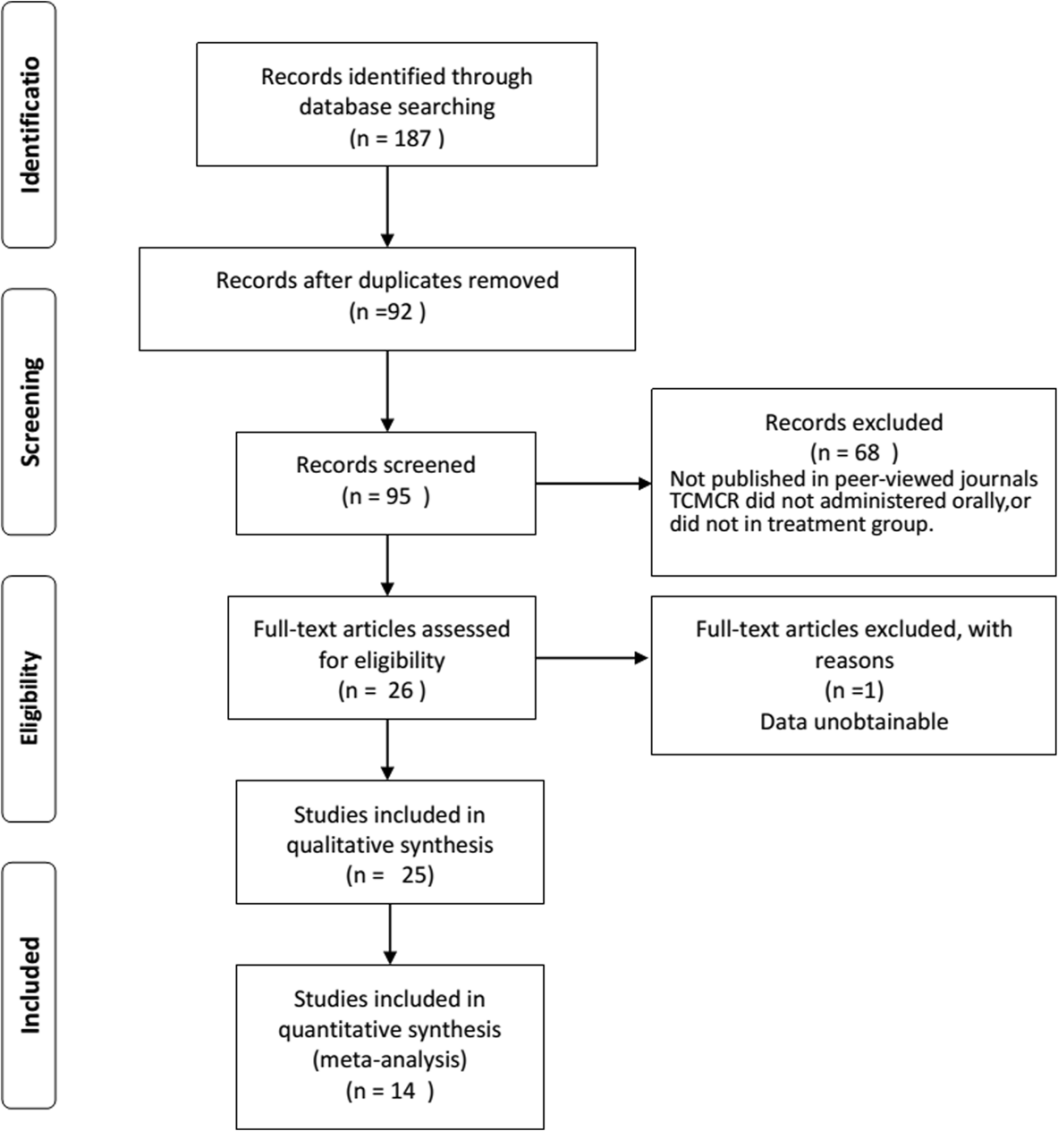
PRISMA flowchart of the study selection

On the basis of predefined standards, we identified 187 potentially relevant articles. After removing duplicate articles, 95 articles remained. Through screening titles and abstracts, 68 were excluded because they were not published in peer-reviewed journals, TCMCR was not administered orally, or traditional Chinese medicine injection/a single Chinese herb medicine was administered in the treatment group. After full-text evaluation of the remaining 26 articles, one article was excluded due to unobtainable data. Thus, 25 studies [7, 8, 19–41] were included for systematic review. Moreover, 14 studies [8, 19, 22–29, 32, 34, 35, 38] were ultimately included in the meta-analysis. Figure 1 shows a flow chart of the study selection.

### Study characteristics

The 25 included studies were all conducted in China and published between 2001 and 2014. Of these, two studies [8, 41] were published in English and 23 others in Chinese. Four studies [22, 30, 34, 39] examined effects in Wistar rats, two [27, 29] in Kunming mice, and 19 in SD rats. The drugs administered to the treatment groups included Naozhenning granules [27], Naochuangning [27, 39], Brain injury compound decoction [37], Tongfujiannao oral liquid [23], Naoshuning [24–26, 32, 35, 38], Shenqi extraction [29], compound Huangjinxiang extraction liquid [40], Quyu Tongfu decoction [40], Angong Niuhuang pill [21, 28], Sanhuang Xiexin decoction [22], Brain Compound decoction [30, 34], Huayu capsules [41], modified “Shengyu” decoction [7, 8], Longxuejie capsules [19], and Jiannao Yizhi capsules [20, 31, 33, 36]. For outcome measurements, brain water content was calculated in 12 studies [8, 22–29, 32, 38], blood–brain barrier permeability was measured in seven studies [24, 25, 27–29, 35, 38], cognitive impairment was evaluated in two trials [37, 39], the TNF-α level was detected in three studies [8, 19, 29] and the NO level was detected in two studies [19, 34]. The characteristics of these studies are listed in Table 3.

**Table 3:** Characteristics of included studies

Study	Animal Species	Treatment Groups(Drug/Dose)	Anaesthetic used	Time of administration	Main Outcomes	Quality Score
Long 2001 [1]	Mixed Kunming mice	①Naozhenning Granule(13.33 g/kg) ②Naochuangning (9.08 g/kg) ③Naochuangning (18.16 g/kg) ④Naochuangning (36.33 g/kg)	Unknown	7 days before injury Daily for 10 days	Cerebral edema BBB permeability	2
Zhang 2002 [3]	SD rats	①Brain injury compound decoction (unknown)	Ether	3 days before injury Daily for 10 days	Independent activity Cognitive performance	4
Wang 2003 [4]	Mixed Wistar rats	①Naozhenning Granule(13.13 g/kg) ②Naochuangning (9.15 g/kg) ③Naochuangning(19.10 g/kg) ④Naochuangning (38.10 g/kg)	Pentobarbital	After injury Daily for 14 days	Memory performance Endothelia content	4
Zhang 2004 [5]	SD rats	①Tongfujiannao Oral Liquid(7.5 g/kg) ②Tongfujiannao Oral Liquid(3.125 g/kg)	Unknown	After injury Daily for 3 days	Brain water content MDA\SOD content(Brain) \LPS content(Plasma) Histopathological	3
Cui 2005a [6]	Male SD rats	①Naoshuning(15 g/kg)	chloral hydrate	3 days before injury Twice for 4 days	Neurological function Histopathological Brain water conte	3
Cui 2005b [7]	Male SD rats	①Naoshuning(15 g/kg)	chloral hydrate	3 days before injury Twice for 4 days	BBB permeability Brain water content MMP-9	3
Yu 2005 [8]	Kunming mice	①Shenqi Extraction (0.28 g/kg) ②Shenqi Extraction (0.14 g/kg) ③Shenqi Extraction (0.07 g/kg)	Ether	3 days before injury Daily for 3 days	BBB permeability Brain water content TNF-α(Brain)\ ET content(Serum)	4
Zhou 2007 [9]	Mixed SD rats	① Huayu capsule (1.030 g/kg) ② Huayu capsule (0.515 g/kg) ③ Huayu capsule (0.258 g/kg)	chloral hydrate	After injury Daily for 7 days	① Nerve-muscle catching capability. ② Histopathological	3
Cui 2008a [10]	Male SD rats	①Naoshuning(7.5 g/kg)	chloral hydrate	3 days before injury Twice for 4 days	Brain water content BBB permeability AQP-4 content	3
Cui 2008b [11]	Male SD rats	①Naoshuning(7.5 g/kg)	Unknown	3 days before injury Twice for 4 days	BBB permeability Histopathological cerebral microvascular density	3
Miao 2008 [12]	Male Wistar rats	①Brain Compound decoction(10 g/kg)	Pentobarbital	Immediately after injury Twice for 7 days	Na^+^-K^+^-ATP Ca^2+^-ATP	5
Cui 2009a [13]	SD rats	①Naoshuning(7.5 g/kg)	Unknown	3 days before injury Daily for 3 days	Histopathological (HE statin) Edema volume Brain water content	3
Cui 2009b [14]	Male SD rats	①Naoshuning(7.5 g/kg)	Unknown	3 days before injury Twice for 8 days	MMP-2/9 content Brain water content EB content	3
Xiong 2009 [15]	SD rats	①Quyu Tongfu decoction(10 ml/kg) ②compound Huangjinxiang extraction liquid(10 ml/kg)	Pentobarbital	20 min after injury Daily for 24 h	AQP- 4 content	5
Xie 2010 [16]	Mixed SD rats	① Angong Niuhuang Pill(0.6 g/kg)	chloral hydrate	12 h before injury Twice for 60 h	Synaptic density BBB permeability Cerebral edema	3
Xu 2010 [17]	Mixed SD rats	①Angong Niuhuang Pill (0.121 g/2 ml)	Pentobarbital	After injury Daily for 12 days	ApoE content (Brain and CSF)	4
Miao 2011 [18]	Male Wistar rats	①Brain Compound decoction(10 g/kg)	Pentobarbital	Immediately after injury Twice for 7 days	NO\nNOS(Brain) Brain water content Histopathological	5
Zhang 2011 [19]	Wistar rats	①Sanhuang Xiexin Decoction(10 g/kg)	chloral hydrate	Immediately after injury Daily for 72 h	NF-κB\IL- 6 content	3
Wang 2012a [20]	SD rats	①Longxuejie capsule (2.6 mg/g/d)	Unknown	after injury Daily for 5d	NO\TNF-α content (serum)	3
Wang 2012b [21]	Male SD rats	①modified “Shengyu”decoction 4.0 mL/200 g/d	Unknown	6 h after injury Twice for 72 h	Histopathological caspase-3 activity	3
Zhao 2012 [22]	Mixed SD rats	①JiannaoYizhi capsules(6.0 g/kg/d)	chloral hydrate	24 h after injury Daily for 10d	CGRP content (Plasma)	3
Zhou 2012 [23]	SD rats	①JiannaoYizhi capsules(6.0 g/kg/d)	chloral hydrate	24 h after injury Daily for 10d	NPY content (serum)	3
Fan 2013a [24]	SD rats	①JiannaoYizhi capsules(6.0 g/kg/d)	chloral hydrate	24 h after injury Daily for 10d	S100B content (serum)	3
Fan 2013b [25]	SD rats	①JiannaoYizhi capsules(6.0 g/kg/d)	chloral hydrate	24 h after injury Daily for 10d	NSE content (serum)	3
Zhao 2014 [26]	Male SD rats	①modified “Shengyu”decoction 0.5 mL/200 g ②modified “Shengyu”decoction 1.0 mL/200 g ③modified “Shengyu”decoction 2.0 mL/200 g	chloral hydrate	6 h after injury Daily for 7d	Neurological function Brain water content Histopathological Lesion volume TNF-α\ IL-1\IL-6\IL-10 content (brain)	6

Note: *BBB* Blood–brain-barrier, *MMP-9* matrix metalloprotein 9, *TNF-α* tumor necrosis factor-α, *IL-1* interleukin-1, *IL-6* interleukin-6, *IL-10* interleukin-10, *NSE* 2-phospho-D-glycerate hydrolase, *NPY* Neuropeptide Y, *CGRP* Calcitonin Gene Related Peptide, *SOD* superoxidedismutase, *MDA* malondialdehyde, *NO* Nitrogen monoxide, *SD* Sprague Dawley.-

### Methodological quality of included studies

Overall, the median quality of the 25 included studies was poor (3, interquartile range, 3–4) with scores ranging from 2 to 6. No studies scored 0 or had a high quality rating (7–10 points). We found one study [8] with a quality score of 6, three [30, 34, 40] with a score of 5, and four [21, 29, 37, 39] with a score of 4. Animals were allocated treatment by randomization in all included articles except one [27]. Only two studies [8, 40] that were included failed to report the monitoring of physiological parameters (although the majority of these only monitored body temperature). All of the studies failed to report potential conflicts of interest, blinded outcome assessment, and blinded induction of TBI.

### Meta-analysis

#### Brain water content

In 12 studies [8, 22–29, 32, 38], there were 25 comparisons (involving 432 animals) of brain water content after TBI, which was determined by the wet and dry weight method [42]. Pooled analysis indicated that animals in the treatment groups had significantly reduced brain water content compared to animals in the control groups (SMD = -1.421, 95 % CI: -1.704 to -1.1379, *P* < 0.0001).

**Fig. 2:**
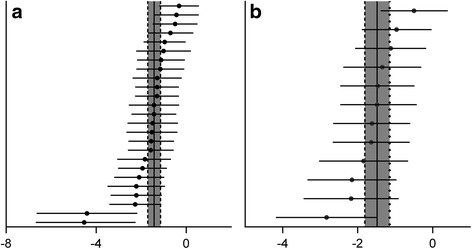
Meta-analysis of effect of TCMCR on brain water content reduction (**a**) and integrity of BBB improvement (**b**). The horizontal lines represent the mean estimated effect size and the 95 % confidence intervals(CI) for each individual comparison according to their effect on brain water content (**a**) and integrity of BBB (**b**). The SMD and the 95 % CI of the global estimate are represented as solid and dashed vertical lines, respectively

**Fig. 3:**
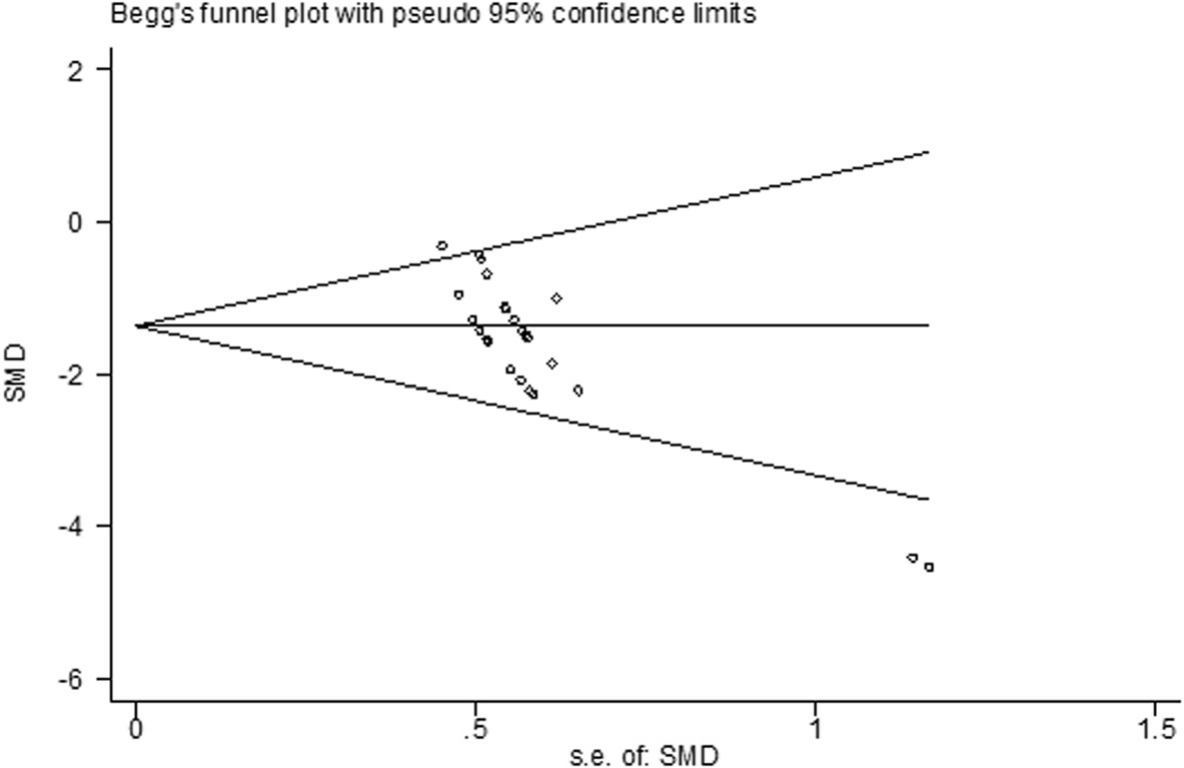
Begg’s funnel plot of brain water content. There was evidence of small study effects (Egger’s test bias coefficient = −5.8081, 95 % CI: −7.7275 to −3.8888, P <0.001)

There was evidence of little heterogeneity among studies (*χ*^2^ = 39.51, df = 24 (*P* = 0.024), *I*^2^ = 39.2 %), and small-study effects (Egger’s test bias coefficient = -5.8081, 95 % CI: -7.7275 to -3.8888, *P* < 0.001). (Figs. 2a and 3)

#### Integrity of blood–brain barrier

In seven included trials [24, 25, 27–29, 35, 38], there were 12 comparisons (involving 224 animals) of blood–brain-barrier integrity after TBI, which was analyzed by assessing extravasation of Evans blue dye [43]. The pooled analysis indicated that animals in the treatment groups had significantly better blood–brain barrier integrity than animals in the control groups (SMD = −1.481; 95 % CI: −1.815 to −1.146; *P* < 0.0001).

**Fig. 4:**
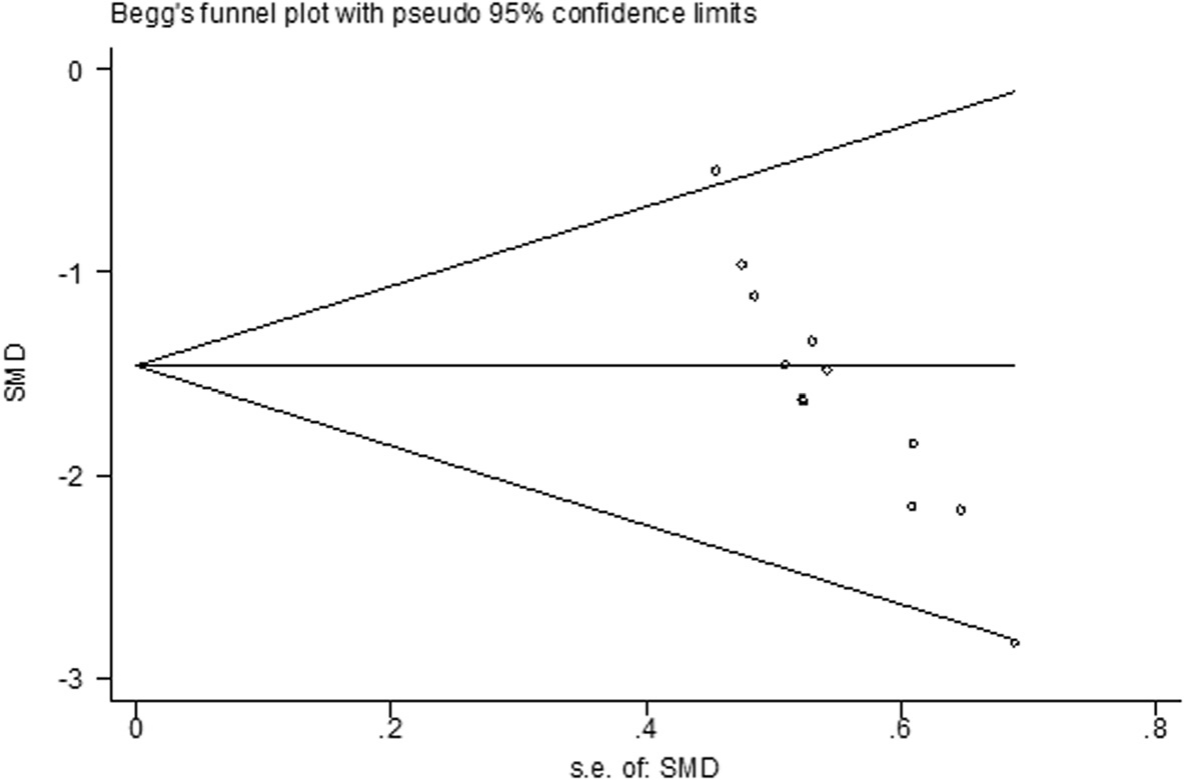
Begg’s funnel plot of integrity of BBB. There was evidence of small study effects (Egger’s test bias coefficient = −8.2850, 95 % CI: −10.3545 to −6.21546, P <0.001)

There was evidence of little heterogeneity among studies (*χ*^2^ = 13.19, df = 11 (*P* = 0.281), *I*^2^ = 16.6 %), and small-study effects (Egger’s test bias coefficient = −8.2850, 95 % CI: −10.3545 to −6.21546, *P* < 0.001). (Figs. 2b and 4)

#### TNF-α levels

In three studies [8, 19, 29], there were seven comparisons (involving 120 animals) of TNF-αafter TBI. The pooled analysis indicated that there was a significant difference in TNF-αlevels between the treatment and control groups (SMD = −1.291; 95 % CI, −1.809 to −0.774; *P* < 0.0001).

**Fig. 5:**
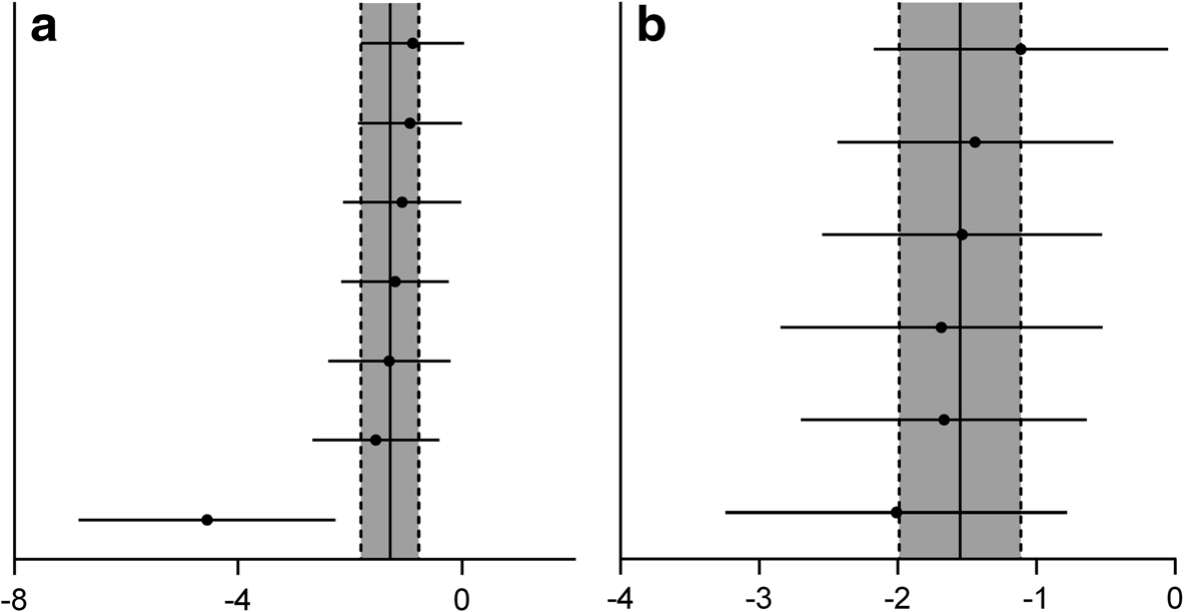
Meta-analysis of effect of TCMCR on the reduction of TNF-α(**a**) and NO(**b**). The horizontal lines represent the mean estimated effect size and the 95 % confidence intervals(CI) for each individual comparison according to their effect on TNF-α(**a**) and NO (**b**). The SMD and the 95 % CI of the global estimate are represented as solid and dashed vertical lines, respectively

There was evidence of little heterogeneity among studies (*χ*^2^ = 9.39, df = 6 (*P* = 0.153), *I*^2^ = 36.1 %). Publication bias could not be assessed because of the small number of studies (<10 studies) [44, 45]. (Fig. 5a)

#### NO levels

In two studies [19, 34], there were six comparisons (involving 108 animals) of NO after TBI. The pooled analysis indicated that there was a significant difference in NO levels between the treatment and control groups (SMD = −1.550; 95 % CI, −1.987 to −1.112; *P* < 0.0001).

There was no evidence of heterogeneity among studies (*χ*^2^ = 1.34, df = 5 (*P* = 0.931), *I*^2^ = 0 %). Publication bias could not be assessed because of the small number of studies (<10 studies) [44, 45]. (Fig. 5b)

### Possible drug protection mechanism analysis

All of the studies selected during initial screening assessed the biological mechanisms of TCMCR activity. A wide variety of possible neuroprotective mechanisms were proposed in these studies. The neuroprotective effect of TCMCR was attributed primarily to preservation of blood–brain barrier integrity, amelioration of cerebral edema, and inhibition of inflammatory response. In addition, it was found that TCMCR may regulate cerebral blood flow. (Table 4)

**Table 4:** Possible protective mechanisms of TCMCR

Possible drug protective mechanisms	Studies
Increase in cerebral microvascular patency and integrity	[11]
Improve cognitive deficits	[3, 4]
Ameliorated cerebral edema	[1, 5–8, 10, 13–16, 19, 26]
Attenuated disruption of the blood–brain-barrier	[1, 7, 8, 10, 11, 14, 16, 24, 25]
Reduced the neuronal apoptosis	[9, 21]
Suppressed oxidative stress	[5, 18, 20]
Regulation of cerebral blood flow	[4, 8, 22, 23]
Increase activities of Na^+^-K^+^-ATPase、Ca^2+^-ATPase and regulation of Ca^2+^	[12]
promote the synthesizing and secreting of apolipoprotein E	[17]
Inhibited the inflammatory response	[5, 8, 19, 20, 26]

## Discussion

To date, numerous clinical trials [46–48] that have sought new therapeutic agents for treating TBI have proven unsuccessful. However, there is increasing evidence that traditional Chinese medicine, including TCMCR, extracts, and acupuncture, have clinical benefit in the treatment of TBI patients [49–51]. Because TCMCR is the main form of TCM drug treatment, robust evidence of its effects on TBI must be provided. Therefore, we have conducted the first systematic review and meta-analysis of the effects of TCMCR in animal models of TBI. Because systematic review and meta-analysis of animal experiments could provide strong evidence in an unbiased manner. Although small-study effects and statistical heterogeneity among studies were present, our results indicated that TCMCR potentially exerts neuroprotective effects in terms of reduction of brain water content, amelioration of BBB permeability, and deduction of TNF-α/NO after TBI. Similar work [52] was performed in the context of experimental stroke that demonstrated the neuroprotective effects of Buyang Huanwu decoction, a well-known TCMCR, on animal stroke models. Though they are different diseases, many aspects of their respective pathologies are similar, and these investigations provide further evidence of the neuroprotective efficacy of TCMCR, supporting its potential use for human TBI therapy.

Concerning study quality, we found that the methodological quality of most included studies was generally poor, as many failed to report blinded outcome assessment, blinded induction of TBI, sample size calculation, compliance with animal welfare regulations, and potential conflicts of interest. However, because we sought to report on overall quality, we did not arbitrarily exclude them solely on the basis of these defects [53].

The current study has some limitations that have also been observed in previous studies [18, 54, 55]. First, although we made an effort to identify all relevant studies, our analysis could only be based on articles published in English or Chinese and did not take the unpublished data and the relevant articles published in other languages into account. Moreover, most of them published in local journal particularly, some publications were showed in journal from university. So there was evidence of small-study effects and publication bias should be considered. Second, no studies specifying the degree of severity (e.g., mild, moderate, or severe). The results of different studies could have been more accurately compared if injury severity had been reported consistently. Third, as in previous studies [56–58], the methodological quality of the included studies was generally poor. Due to the poor quality of the studies, the results of this review are likely to be influenced by many factors. Of course, it should be noted that negative judgment did not necessarily indicate that the experiment itself was performed inadequately; it indicated that there was inadequate information for assessing its quality. Fourth, although the findings indicate that TCMCR treatment benefits can be found in the TBI model with weight-drop mothed. For the weight-drop model, this model is limited in the production of primary lesions that are macroscopically meaningful once it is not capable of creating cranial fractures with levels of energy compatible with life after impact. It presented discrete focal lesions in a small number of animals and only at elevated levels of energy, but in accordance with what was described in similar studies [59]. Moreover, any single animal model may not be able to fully mimic the highly heterogeneous nature of human TBI [60]. Lastly, we limited our analysis to the alternation of BBB integrity, brain water content, and TNF-α/NO levels following TBI, largely due to insufficient data regarding histopathology, such as lesion volume and neurobehavioral outcomes such as cognitive performance and motor function.

Additionally, heterogeneity must be considered for any meta-analysis. The main reasons for heterogeneity were the limited number of trials and small cohorts [61]; therefore, additional large-scale clinical trials are required. Another important reason for the existence of heterogeneity was the low quality and potential bias of the trials selected for analysis. The surprisingly low heterogeneity of NO levels in the meta-analysis requires further consideration. It is also associated with the poor methodological quality of the selected trials, which require additional investigation.

To improve the clinical translation, our recommendations for the conduct of future animal studies of TCMCR or other TCM drugs are as follows: (1) More studies have shown that TCMCR is a whole greater than the sum of its parts in terms of its composition and pharmacodynamic action. In order to make full use of the advantages offered by TCMCR, it is essential to address the difficulties in studying TCMCR, including the role played by the material basis and physical basis in TCM’s therapeutic effects, and the rules of compatibility, pharmacology, and action mechanism of TCMCR [62]; (2) Other TBI models are needed to investigate the effects of TCM/TCMCR on TBI; (3) Further researchers are strongly recommended to consult and follow the ARRIVE (Animal Research: Reporting In Vivo Experiments) guidelines to report their animal experimental results [63, 64]; (4) Other long-term neuropsychological outcomes should also be focused on, such as the cognitive performance, the motor function.

## Conclusions

Despite limitations, the animal data has shown that TCMCR may be neuroprotective in the TBI model with weight-drop mothed. However, successful clinical translation of this neuroprotective strategy necessitates rigorous, robust, and detailed pre-clinical evaluation. Therefore, additional well-designed and well-reported experimental animal studies are needed.
